# A randomized, open-label, multicenter, phase II study evaluating the efficacy and safety of BTH1677 (1,3–1,6 beta glucan; Imprime PGG) in combination with cetuximab and chemotherapy in patients with advanced non-small cell lung cancer

**DOI:** 10.1007/s10637-017-0450-3

**Published:** 2017-03-16

**Authors:** M. Thomas, P. Sadjadian, J. Kollmeier, J. Lowe, P. Mattson, J. R. Trout, M. Gargano, M. L. Patchen, R. Walsh, M. Beliveau, J. F. Marier, N. Bose, K. Gorden, F. Schneller

**Affiliations:** 1 Internistische Onkologie der Thoraxtumoren, Thoraxklinik im Universitätsklinikum Heidelberg, Translational Lung Research Center Heidelberg (TLRC-H), Member of the German Center for Lung Research (DZL), Amalienstrasse 5, 69126 Heidelberg, Germany; 2 Johannes Wesling Medical Center Minden, Clinic Hematology/Oncology, Hans-Nolte-Str. 1, 32429 Minden, Germany; 3 Lungenklinik Heckeshorn, HELIOS Klinikum Emil von Behring, Specialist Department 1: Clinic for Pneumology, Pneumology Oncology, Walterhöferstr.11, 14165 Berlin, Germany; 4 Biothera Pharmaceuticals Inc., 3388 Mike Collins Drive, Suite A, Eagan, MN 55121 USA; 5 Rutgers University, 82 Rittenhouse Circle, Newtown, PA 18940 USA; 6 Immuno Research, Inc., 3388 Mike Collins Drive, Suite B, Eagan, MN 55121 USA; 7 Pharsight/Certara, Pharsight – A Certara™ Company, 2000 Peel Street, Suite 570, Montréal, Québec, H3A 2W5 Canada; 8 Medical Clinic and Polyclinic, Klinikum rechts der Isar of Technical University Munich, Ismaninger Str. 22, 81675 Munich, Germany

**Keywords:** Immunotherapy, NSCLC, Cetuximab, Biomarker, Beta glucan

## Abstract

*Introduction* BTH1677, a 1,3–1,6 beta-glucan immunomodulator, stimulates a coordinated anti-cancer immune response in combination with anti-tumor antibody therapies. This phase II study explored the efficacy, pharmacokinetics (PK), and safety of BTH1677 combined with cetuximab/carboplatin/paclitaxel in untreated stage IIIB/IV non-small cell lung cancer (NSCLC) patients. *Methods* Patients were randomized 2:1 to the BTH1677 arm (*N*=60; BTH1677, 4 mg/kg, weekly; cetuximab, initial dose 400 mg/m^2^ and subsequent doses 250 mg/m^2^, weekly; carboplatin, 6 mg/mL/min AUC (area-under-the-curve) by Calvert formula, once each 3-week cycle [Q3W]); and paclitaxel, 200 mg/m^2^, Q3W) or Control arm (*N*=30; cetuximab/carboplatin/paclitaxel as above). Carboplatin/paclitaxel was discontinued after 4–6 cycles; patients who responded or remained stable received maintenance therapy with BTH1677/cetuximab (BTH1677 arm) or cetuximab (Control arm). Investigator and blinded central radiology reviews were conducted. Efficacy assessments included objective response rate (ORR; primary endpoint), disease control rate, duration of objective response, time-to-progression and overall survival (OS); safety was assessed by adverse events (AEs). Potential biomarker analysis for BTH1677 response was also conducted. *Results* Compared to control treatment, the addition of BTH1677 numerically increased ORR by both investigator (47.8% vs 23.1%; p=0.0468) and central (36.6% vs 23.1%; p=0.2895) reviews. No other endpoints differed between arms. PK was consistent with previous studies. BTH1677 was well tolerated, with AEs expected of the backbone therapy predominating. Biomarker-positive patients displayed better ORR and OS than negative patients. *Conclusions* BTH1677 combined with cetuximab/carboplatin/paclitaxel was well tolerated and improved ORR as first-line treatment in patients with advanced NSCLC. Future patient selection by biomarker status may further improve efficacy

**ClinicalTrials.gov** Identifier: NCT00874848

## Introduction

Lung cancer is the second most commonly diagnosed cancer in both men and women after prostate and breast cancer, respectively [1]. Approximately 85% of all lung cancers consist of non-small cell lung cancer (NSCLC) and patients usually present with locally advanced or metastatic disease at initial diagnosis [1]. For years, the first-line standard of care treatment for advanced NSCLC consisted of platinum-based combination chemotherapies [2, 3]. Although still a mainstay therapy for many NSCLC patients [4], advances in understanding the molecular pathways driving carcinogenesis (e.g., epidermal growth factor receptor [EGFR] gene mutations and anaplastic lymphoma kinase [ALK] translocations) led to development of targeted EGFR tyrosine kinase inhibitors and ALK-directed therapies that proved superior to chemotherapies for first-line management of advanced disease in select molecularly-defined patient subgroups harboring mutations sensitive to these therapies [4, 5]. Several monoclonal antibody (MAb) therapies have also been developed for first-line treatment of advanced NSCLC [4], including bevacizumab, which targets vascular endothelial growth factor (VEGF) [6], cetuximab [7] and necitumumab [8], both of which target EGFR and, most recently, pembrolizumab, which targets the programmed death-1 (PD-1) immune checkpoint receptor on cytotoxic T-cells [9]. Additional MAbs approved for second-line therapy, but likely to soon be front-line therapy, include ramucirumab, an agent targeting VEGF receptor 2 (VEGFR2) [10], as well as the anti-PD-1 and anti-programmed death ligand-1 (PD-L1) MAbs, nivolumab [11] and atezolizumab [12]. Unlike the other MAbs mentioned above, cetuximab ultimately never achieved regulatory approval for treatment of NSCLC. However, results of phase III NSCLC studies with cetuximab in combination with platinum doublet chemotherapy did show modest improvements in clinical efficacy endpoints [13, 14], which led to National Comprehensive Cancer Network guidelines recommending treatment of NSCLC with regimens containing cetuximab in select patients [4].

BTH1677 (β(1,6)-[poly-(1,3)-D-glucopyranosyl]-poly-β-(1,3)-D-glucopyranose; Imprime PGG; Biothera Pharmaceuticals Inc., Eagan, MN), is a fungal-derived, water-soluble, 1,3–1,6 beta glucan. It is purified from the cell wall of a proprietary, non-recombinant, strain of the yeast *Saccharomyces cerevisiae* and consists purely of D-glucose molecules joined together via beta 1,3 and 1,6 linkages. Specifically, it consists of a backbone of 1,3 linked glucose residues with periodic branches linked to the backbone in a 1,6 configuration. Similar to the backbone, the side chains also contain multiple 1,3-linked glucose residues. No beta glucans have been approved for clinical use in the United States. However, in China and Japan, two fungal mushroom-derived 1,3–1,6 beta glucans (lentinan and schizophyllan) have been approved. The chemical structure of BTH1677 is different from that of mushroom-derived beta glucans which have a higher branching frequency and side chains that consist of only single glucose residues [15–17].

BTH1677 functions as a pathogen-associated molecular pattern (PAMP) molecule to stimulate a coordinated innate and adaptive anti-cancer immune response in combination with anti-tumor antibody therapies. When BTH1677 enters the blood, it is bound by endogenous plasma anti-beta glucan antibodies (ABA) resulting in complement activation and opsonization with complement protein iC3b [18, 19]. The BTH1677/ABA/iC3b complex initially binds to innate immune effector cells through complement receptor 3 and Fc gamma receptor IIA (FcγIIA) [18, 19], activating innate immune cell function and enabling direct killing of antibody-targeted tumor cells [18]. BTH1677 also enables reeducation of the tumor microenvironment, shifting the normally suppressive M2-state macrophages to a more M1 (tumor attack) state [20, 21], and promoting depletion and/or maturation of myeloid-derived suppressor cells in the tumor microenvironment [22]. BTH1677 treatment additionally activates antigen-presenting cells, driving co-stimulatory marker expression on macrophages and dendritic cells, as well as dendritic cell maturation, CD4 and CD8 T-cell expansion, and production of key anti-tumor cytokines (e.g., interferon gamma) [21, 23–26]. In murine syngeneic and xenogeneic tumor models, the administration of BTH1677 with various tumor-targeting monoclonal antibodies has resulted in greater suppression of tumor growth and longer survival than with either agent alone [27–29]. One of these studies [28] specifically evaluated BTH1677 and cetuximab in a lung cancer model. Similar effects have been observed with BTH1677 combined with anti-angiogenic antibodies (anti-VEGF and anti-VEGFR2) [22, 30, 31] and checkpoint inhibitor antibodies [32, 33].

Clinically, in healthy subjects, BTH1677 was well tolerated after single doses up to 6 mg/kg and after 7 daily doses up to 4 mg/kg and pharmacokinetic (PK) parameters demonstrated linearity with dose [34]. Additionally, BTH1677 in combination with cetuximab, with or without irinotecan, was well tolerated with promising evidence of efficacy in a phase Ib/II study in patients with recurrent or progressive metastatic colorectal cancer (mCRC) [35–37].

Based on the preclinical efficacy observed in vivo in murine lung cancer models with BTH1677 combined with either cetuximab [28] or bevacizumab [31], parallel studies were designed to evaluate these BTH1677 combinations in NSCLC patients. Here we report results of the randomized, open-label, multicenter, phase II study (NCT00874848) designed to evaluate the antitumor activity, safety, and PK profile of BTH1677 when combined with cetuximab and concomitant carboplatin and paclitaxel therapy in patients with previously untreated, advanced NSCLC. Exploratory evaluation of the potential use of baseline ABA levels as a measure of a patient’s ability to respond to BTH1677 is also presented. At the time this study was initiated, cetuximab was undergoing regulatory review for first-line use in late-stage NSCLC patients, with approval anticipated. This anticipation, along with our preclinical and clinical mCRC data demonstrating enhanced antitumor effects with BTH1677 combined with cetuximab therapy prompted us to evaluate this combination in NSCLC. Although cetuximab was ultimately not approved and is not currently a standard therapy for NSCLC, the results of this study nevertheless support the concept of improved efficacy with the addition of BTH1677 to antitumor antibody therapy.

## Materials and methods

### Study objectives

The primary objective was to evaluate the objective response rate (ORR; complete response [CR] + partial response [PR]) in each treatment arm. Secondary objectives included assessment of best response rate (CR, PR, and stable disease [SD] rates), disease control rate (DCR; CR + PR + SD), duration of objective tumor response (DOR), time-to-progression (TTP), and overall survival (OS) in each treatment arm. Safety within each arm and the PK profile of BTH1677 (BTH1677 arm only) were also evaluated. Exploratory analysis evaluated correlations between baseline ABA levels and clinical responses.

### Patient eligibility

Patients, 18 to 75 years of age, provided written informed consent, and had histologically or cytologically confirmed stage IIIB or IV NSCLC according to American Joint Committee on Cancer (AJCC) Staging v6; measurable disease as defined by Response Evaluation Criteria in Solid Tumors (RECIST) v1.0; Eastern Cooperative Oncology Group (ECOG) performance status (PS) 0 or 1; life expectancy of >3 months; adequate hematologic, renal, and hepatic function; and use of an effective contraceptive.

Exclusion criteria included prior systemic chemotherapy for lung cancer; previous radiation therapy to >30% of active bone marrow or any radiation therapy within 3 weeks of study Day 1; central nervous system metastases; uncontrolled hypertension; peripheral neuropathy ≥Grade 2; fever >38.5 °C within 3 days of Day 1; active yeast infection; human immunodeficiency virus/acquired immune deficiency syndrome, hepatitis B, or hepatitis C; connective tissue or autoimmune disease; previous organ or progenitor/stem cell transplant; history of myocardial infarction or any other unstable or uncontrolled heart disease; second malignancy within the previous 5 years (other than basal cell carcinoma, cervical intra-epithelial neoplasm, or curatively treated prostate cancer); known hypersensitivity to baker’s yeast, murine proteins, or Cremophor® EL; previous exposure to cetuximab or BTH1677; or investigational therapy within 30 days prior to Day 1. Females were also excluded if they were pregnant or breastfeeding.

### Study design and treatment plan

This randomized, open-label, multicenter phase II study was performed at sites in Germany and the United States and was conducted in full accordance with the Good Clinical Practice: Consolidated Guideline approved by the International Conference on Harmonisation and all other applicable national and local laws/regulations. All study materials were approved by the governing ethics committee or institutional review board at each site.

The study tested the null hypothesis that the true ORR was ≤30% vs the alternative hypothesis that the true ORR in the BTH1677 arm was at least 50%. Based on the Simon 2-stage optimal flexible design [38] it was determined that 60 patients in the BTH1677 arm would provide 90% power for the hypotheses testing at an alpha level of 5%. With 2:1 randomization, a sample size of 30 patients was determined for the Control arm. The first stage enrolled 15 and 7 evaluable patients in the BTH1677 and Control arms, respectively. Criteria for progression to the second stage were met and enrollment continued to the full planned patient numbers.

Patients were dosed in 3-week cycles. On Days 1, 8, and 15 of each cycle, patients in the BTH1677 arm were administered 4 mg/kg of BTH1677 (intravenous [IV] over 2 to 4 h, depending on patient weight and total dose administered). On these same days, cetuximab was administered to all patients in both arms (initial IV loading dose of 400 mg/m^2^ over 120 min and subsequent IV doses at 250 mg/m^2^ over 60 min); in the BTH1677 arm, cetuximab was administered after BTH1677. On Day 2 of each cycle, IV carboplatin (dosed according to Calvert formula area under the curve [AUC] of 6 mg/mL/min over 30 min) and IV paclitaxel (200 mg/m^2^ over 3 h) were administered to all patients. Prior to each BTH1677 dosing, all patients were to receive low-dose corticosteroids and a histamine-1 antagonist (e.g., 4 mg of dexamethasone orally [PO] and 50 mg of diphenhydramine IV). On Day 2 of each cycle, all patients were pre-medicated with the local clinic’s regimen of corticosteroids and antihistamines prior to carboplatin and paclitaxel therapy. Premedication for the Control arm patients was performed according to local clinical practice for cetuximab.

Carboplatin and paclitaxel administration continued for at least four cycles, but could continue for up to six cycles at the investigator’s discretion. Following completion of their sixth dosing cycle, patients who experienced a response (CR or PR) or had remained stable (SD) were eligible to continue on maintenance therapy receiving BTH1677 and cetuximab (BTH1677 arm) or cetuximab alone (Control arm).

### Study assessments

Safety and tolerability were assessed by adverse events (AEs; National Cancer Institute Common Terminology for Adverse Events [CTCAE] v3.0), physical examinations, and laboratory tests.

Tumor response assessments were based on computed tomography (CT) scans performed every other cycle (i.e., at 6-week intervals). Both investigator and blinded central independent radiology reviews were performed. Tumor response was assessed using a modified RECIST v1.0 criteria in which an initial response did not require a repeat assessment for confirmation. All other RECIST v1.0 criteria remained unmodified.

ABA assessments were performed by prototype enzyme-linked immunosorbent assays (ELISAs) developed at Biothera Pharmaceuticals Inc. to detect serum IgG and IgM ABA [18, 39]. A commercially available pooled human serum was assigned an arbitrary value of IgG or IgM ABA units per milliliter (relative antibody units/mL; RAU/mL) and run as a standard curve in each assay to determine test sample ABA levels. A previous study had shown correlations between ABA levels and the ability of BTH1677 to activate complement (as measured by C5a production), bind to neutrophils, induce neutrophil surface expression of complement receptor 3 (CR3), modulate neutrophil activation markers (CD88 and CD62), and induce production of interleukin-8 (IL-8) when incubated with whole blood samples obtained from healthy subjects [18, 39]. These effects were consistently seen in blood with ABA levels above the thresholds of 235 IgG RAU/mL or 330 IgM RAU/mL, but were not seen, or seen at much lower levels, in blood with ABA levels below these thresholds [18, 39]. Subjects with ABA levels above the thresholds were identified as biomarker positive and those below the thresholds were identified as biomarker negative. These prospectively defined thresholds from the healthy volunteer study were applied to the NSCLC patient samples to determine patient ABA biomarker status (ie, positive vs negative). At the time of this NSCLC study, the importance of ABA in the mechanism of action of BTH1677 was not known and the protocol did not include collection of samples for ABA analysis. However, it was possible to perform ABA determinations from serum samples remaining from baseline (pre-dosing) PK samples. Since PK samples were only collected from patients in the BTH1677 arm, biomarker analyses could be performed only in these patients. Correlation of ABA status and clinical responses was performed in post-hoc hypotheses-generating analyses.

### Pharmacokinetics

PK assessments for BTH1677 were based on post-dosing blood samples collected on Day 1 of Cycles 1 and 3. Samples for BTH1677 trough level assessments were also obtained before dosing on all other weeks of Cycles 1 and 2. Serum BTH1677 levels were measured by a beta-glucan specific ELISA developed at Biothera Pharmaceuticals Inc. which had a lower limit of detection of 1.2 ng/mL and a limit of quantitation of 4.7 ng/mL. Serum PK parameters of BTH1677 were calculated using noncompartmental analysis (NCA) with NCA model 202 (constant infusion dose input) in WinNonlin® v5.2.

### Statistical analysis

**Fig. 1:**
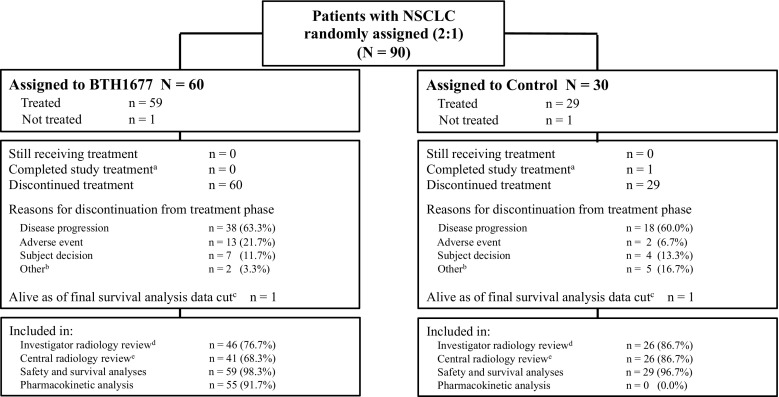
Patient Disposition. ^a^Per protocol, completion of treatment was defined as patients completing 18 cycles of treatment without progressive disease; ^b^For the BTH1677 arm these included lost to follow-up (*n* = 1) and never treated (*n* = 1); For the Control arm these included investigator decision (*n* = 2), noncompliance (*n* = 2), and never treated (*n* = 1); ^**c**^Final survival analysis was performed approximately 4 years after the randomization date of the last patient enrolled into study; ^d^Reasons for exclusion from efficacy analyses related to investigator radiology review in the BTH1677 arm were no evaluable post-baseline CT scan (*n* = 14; none of these patients had a best response of disease progression ie, clinical progression, reported by the investigator); in the Control arm reasons were no evaluable baseline and/or post-baseline CT scan (*n* = 4; none of these patients had a best response of disease progression (ie, clinical progression) reported by the investigator); ^e^Reasons for exclusion from efficacy analyses related to central radiology review in the BTH1677 arm were no evaluable baseline and/or post-baseline CT scan (*n* = 19; one of these patients had a best response of disease progression ie, clinical progression, reported by the investigator); in the Control arm reasons were no evaluable baseline and/or post-baseline CT scan (*n* = 4; none of these patients had a best response of disease progression (ie, clinical progression) reported by the investigator)

The analysis populations for investigator and central radiology tumor assessments, safety and survival analyses, and PK analyses are shown by treatment arm in Fig. 1. The analysis populations for tumor assessments (primary efficacy populations) were comprised of all randomized patients who received any amount of cetuximab, carboplatin, or paclitaxel, with or without BTH1677, and who had an evaluable baseline CT scan assessment and at least one evaluable post-baseline CT scan assessment. The investigator and central radiology review populations were not identical due to separate assessments of the above criteria by the two review groups. The safety and survival populations were comprised of all randomized patients who received any amount of study drugs. The PK population was comprised of all patients who had at least 75% of the PK measurements available for any particular treatment cycle data set.

Efficacy and safety measures are displayed by treatment arm. Categorical data are presented by *n* and % for each category and continuous data are presented by mean and standard deviation (SD). Kaplan-Meier estimates were utilized for time-to-event analyses and, where appropriate, 95% confidence intervals (CI) are provided. Comparisons between treatment arms were performed at a 0.05 level of significance. AEs are summarized by system organ class using the Medical Dictionary for Regulatory Activities v15.0.

## Results

### Patient disposition

Between August 2009 and November 2012, 90 patients with NSCLC were randomized 2:1 to the BTH1677 arm or the Control arm. As shown in Fig. 1, a total of 88 patients (BTH1677, *N* = 59; Control, *N* = 29) were treated and included in the safety and survival analyses. The investigator radiology review population included 46 patients in the BTH1677 arm and 26 patients in the Control arm. The central radiology review population included 41 patients in the BTH1677 arm and 26 patients in the Control arm. The primary reason for patient exclusion from either review population was the absence of baseline or post-baseline CT scan data. The primary reason for treatment discontinuation in each arm was tumor progression (BTH1677, 63.3%; Control, 60.0%).

Patient demographics and disease characteristics at baseline are shown in Table 1. The BTH1677 and Control arms were generally similar with regard to race, age, ECOG PS, disease stage at initial diagnosis, and time from initial tumor diagnosis. There were more males in the BTH1677 arm (74.6%) than in the Control arm (58.6%) and the percentage of patients who received prior radiotherapy was higher in the Control arm (10.3%) than in the BTH1677 arm (0%).

**Table 1:** Patient demographics and disease characteristics at baseline (Safety population)

Characteristic	BTH1677 (*N* = 59)	Control (*N* = 29)
Age (years)		
Median (range)	58 (38, 78)	65 (41, 71)
Sex, n (%)		
Male	44 (74.6)	17 (58.6)
Female	15 (25.4)	12 (41.4)
Race, n (%)		
White	56 (94.9)	29 (100.0)
Black	2 (3.4)	0
Other	1 (1.7)	0
ECOG performance status, n (%)		
0	20 (33.9)	10 (34.5)
1	38 (64.4)	18 (62.1)
Missing	1 (1.7)	1 (3.4)
Disease stage at initial diagnosis, n (%)^a^		
IIIB	3 (5.1)	4 (13.8)
IV	56 (94.9)	25 (86.2)
Histology type, n (%)		
Squamous	16 (27.1)	11 (37.9)
Non-squamous	42 (71.2)	18 (62.1)
Missing	1 (1.7)	0
Time from initial tumor diagnosis (months)^b^		
Median (range)	0.7 (0.2, 6.5)	0.5 (0, 8.2)^c^
Prior cancer treatment, n (%)		
Radiotherapy	0	3 (10.3)
Surgery	23 (39.0)	13 (44.8)
Chemotherapy^d^	1 (1.7)	1 (3.4)

*ECOG* Eastern Cooperative Oncology Group

^a^At the time this study was performed, malignant pleural or pericardial effusions were considered Stage IIIB, however as of the 7th edition of the Cancer Staging Manual of the American Joint Committee on Cancer this would be considered Stage IV (ie, by current staging, all patients were Stage IV)

^b^Time from initial tumor diagnosis (months) = screening visit – initial tumor diagnosis date)/30

^c^Based on *N* = 28

^d^One patient in the BTH1677 arm received prior chemotherapy for Non-Hodgkin’s lymphoma and 1 patient in the Control arm received radiotherapy that was inadvertently categorized as chemotherapy

#### Efficacy

##### Tumor-associated assessments

Tumor response assessments are shown in Table 2. For both investigator and central radiology reviews, ORR for the BTH1677 arm was numerically increased; however, statistical significance was seen only in the investigator review. Investigator review ORR for the BTH1677 and Control arms, respectively, was 47.8% (95% CI: 32.9, 63.1) and 23.1% (95% CI: 9.0, 43.6) (*p* = 0.0468) and central review ORR for the BTH1677 and Control arms, respectively, was 36.6% (95% CI: 22.1, 53.1) and 23.1% (95% CI: 9.0, 43.6) (*p* = 0.2895). In both reviews, all responses were PR. Taking into account the SD rates, DCRs were high in both reviews and did not statistically differ between treatment arms in either review (investigator BTH1677 78.3% [95% CI 63.6, 89.1] vs Control 76.9% [95% CI 56.4, 91.0], *p* = 1.000; central BTH1677 85.4% [95% CI 70.8, 94.4] vs Control 80.8% [95% CI 60.6, 93.4], *p* = 0.7385). The DOR and TTP also did not differ between treatment arms in either review. DOR and TTP for the BTH1677 arm vs Control arm by the investigator review were 3.8 (95% CI: 2.8, 4.2) vs 4.7 (95% CI: 1.4, not estimable) months and 4.3 (95% CI: 3.6, 5.6) vs 4.4 (95% CI: 3.2, 5.9) months, respectively, and by the central review were 4.4 (95% CI: 2.8, 6.5) vs 4.1 (95% CI: 1.4, 5.4) months and 6.4 (95% CI: 4.3, 8.3) vs 6.0 (95% CI: 4.3, 7.1) months, respectively.

**Table 2:** Tumor-associated assessments based on investigator and central radiology reviews (Primary efficacy populations)

	Investigator radiology review					Central radiology review				
	BTH1677 (*N* = 46)		Control (*N* = 26)			BTH1677 (*N* = 41)		Control (*N* = 26)		
	n (%)	95% CI	n (%)	95% CI	*P* value	n (%)	95% CI	n (%)	95% CI	*P* value
Objective response rate	22 (47.8)	(32.9, 63.1)	6 (23.1)	(9.0, 43.6)	0.0468	15 (36.6)	(22.1, 53.1)	6 (23.1)	(9.0, 43.6)	0.2895
Disease control rate	36 (78.3)	(63.6, 89.1)	20 (76.9)	(56.4, 91.0)	1.000	35 (85.4)	(70.8, 94.4)	21 (80.8)	(60.6, 93.4)	0.7385
Best observed response										
Complete response	0	0	0	0		0	0	0	0	
Partial response	22 (47.8)	(32.9, 63.1)	6 (23.1)	(9.0, 43.6)		15 (36.6)	(22.1, 53.1)	6 (23.1)	(9.0, 43.6)	
Stable disease	14 (30.4)	(17.7, 45.8)	14 (53.8)	(33.4, 73.4)		20 (48.8)	(32.9, 64.9)	15 (57.7)	(36.9, 76.6)	
Progressive disease	10 (21.7)	(10.9, 36.4)	6 (23.1)	(9.0, 43.6)		6 (14.6)	(5.6, 29.2)	5 (19.2)	(6.6, 39.4)	
Duration of objective tumor response	BTH1677 (*N* = 22)		Control (*N* = 6)		HR (95% CI)^a^/ Log-rank *P* value	BTH1677 (*N* = 15)		Control (*N* = 6)		HR (95% CI)^a^/ Log-rank *P* value
Number of patients (%) with objective response (CR + PR)	22 (100.0)		6 (100.0)			15 (100.0)		6 (100.0)		
Number of patients (%) with known duration (uncensored)	18 (81.8)		5 (83.3)			8 (53.3)		3 (50.0)		
Number of patients (%) with unknown duration (censored)	4 (18.2)		1 (16.7)			7 (46.7)		3 (50.0)		
Duration of objective response (months)^b^										
Median (95% CI)	3.8 (2.8, 4.2)		4.7 (1.4, NE)		1.563 (0.574, 4.253) 0.3670	4.4 (2.8, 6.5)		4.1 (1.4, 5.4)		0.594 (0.148, 2.382) 0.4456
Time-to-Progression	BTH1677 (*N* = 46)		Control (*N* = 26)		HR (95% CI)^a^ Log-rank *P* value	BTH1677 (*N* = 41)		Control (*N* = 26)		HR (95% CI)^a^ Log-rank *P* value
Number of patients with progressive disease, n (%)	38 (82.6)		21 (80.8)			20 (48.8)		14 (53.8)		
Number of patients censored, n (%)	8 (17.4)		5 (19.2)			21 (51.2)		12 (46.2)		
Time-to-progression (months)^c^										
Median (95% CI)	4.3 (3.6, 5.6)		4.4 (3.2, 5.9)		1.10 (0.65, 1.90) 0.7305	6.4 (4.3, 8.3)		6.0 (4.3, 7.1)		0.83 (0.42, 1.69) 0.6044

Tumor response data reported as the number (n) and percent (%) of patients and the 95% exact binomial confidence interval

*CI* confidence interval, *CR* complete response, *PR* partial response, *HR* hazard ratio, *NE* not estimable

^a^Hazard ratio and 95% CI based on Cox proportional hazards model with treatment as a factor

^b^Duration of objective response (months) based on Kaplan-Meier estimates

^c^Time-to-progression (TTP) (months) based on Kaplan-Meier estimates. TTP was defined as the time from the date of randomization to the first date of documented progressive disease. Patients who died on study from other causes (not related to study disease) and patients who were lost to follow up or who were alive without documented progressive disease as of the data cut-off date for the analysis were censored at the last tumor assessment date

##### Overall survival

**Fig. 2:**
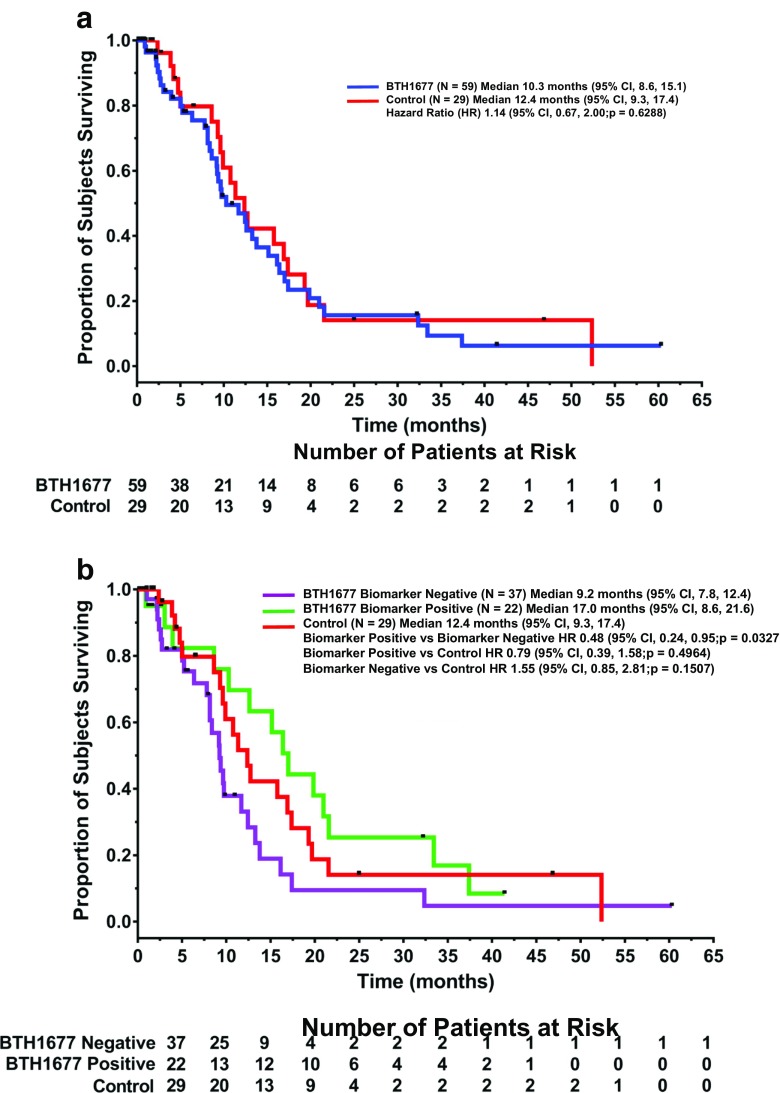
Overall Survival. The Kaplan-Meier overall survival curves from patients in the safety population are shown by treatment arm (Fig. 2a) and by anti-beta-glucan antibody biomarker status (Fig. 2b). HR, hazard ratio; CI, confidence interval

The OS Kaplan Meier curves for the BTH1677 and Control arms are shown in Fig. 2a. The median OS of patients in the BTH1677 arm was 10.3 months (95% CI: 8.6, 15.1) compared with 12.4 months (95% CI: 9.3, 17.4) in the Control arm. No statistical significance in OS was observed between arms (hazard ratio 1.14; 95% CI: 0.67, 2.00; *p* = 0.6288).

##### Exploratory analyses by ABA biomarker status

Of the 59 BTH1677 patients in the study, 22 (37.3%) were determined to be ABA biomarker positive and 37 (62.7%) to be ABA biomarker negative. In the radiology review populations, there were 15 biomarker-positive and 31 biomarker-negative patients in the investigator review population and 13 biomarker-positive and 28 biomarker-negative patients in the central review population. Based on the investigator review, compared to ORRs of 23.1% in the Control arm and 47.8% in the BTH1677 arm, ORR in the biomarker-negative BTH1677 patients was 38.7% (95% CI: 21.8, 57.8; *p* = 0.2592 vs Control) and 66.7% in the biomarker-positive BTH1677 patients (95% CI: 38.4, 88.2; *p* = 0.0088 vs Control; *p* = 0.1162 vs BTH1677 biomarker negative). However, based on central review, compared to ORRs of 23.1% in the Control arm and 36.6% in the BTH1677 arm, ORR in the biomarker-negative BTH1677 patients was 35.7% (95% CI: 18.6, 55.9; *p* = 0.3791 vs Control) and 38.5% in the biomarker-positive BTH1677 patients (95% CI: 13.9, 68.4; *p* = 0.4528 vs Control; *p* = 1.000 vs BTH1677 biomarker negative). Survival favored BTH1677 biomarker-positive patients (Fig. 2a). Compared to median OS of 12.4 months in the Control arm and 10.3 months in the BTH1677 arm, median OS in the biomarker-negative BTH1677 patients was 9.2 months (vs Control, HR 1.55 [95% CI: 0.85, 2.81]; *p* = 0.1507) and 17.0 months in the biomarker-positive BTH1677 patients (vs Control HR 0.79 [95% CI: 0.39, 1.58]; *p* = 0.4964; vs biomarker negative, HR 0.48 [95% CI: 0.24, 0.95]; *p* = 0.0327).

### Safety

All patients receiving any treatment (BTH1677 *N* = 59; Control *N* = 29) were included in the safety population and all of these patients experienced at least 1 AE (Table 3). In the BTH1677 arm, 44.1% and 28.8% of the patients were assessed as having AEs that were probably or possibly related to BTH1677, respectively. Grade 3 or Grade 4 AEs occurred at a slightly lower incidence in the BTH1677 arm vs Control arm (78.0% vs 86.2%). However, serious adverse events (SAEs) and AEs leading to treatment discontinuation occurred at a higher incidence in the BTH1677 arm vs the Control arm (62.7% vs 41.4% and 22.0% vs 6.9%, respectively). SAEs occurring in more than one patient in the Control arm were neutropenia, diarrhea, and pulmonary embolism (each occurring in 2 patients [6.9%]). SAEs occurring in more than one patient in the BTH1677 arm were pleural effusion (6 patients [10.2%]), pulmonary embolism (5 patients [8.5%]), and dyspnea and hemoptysis (each occurring in 2 patients [3.4%]). AEs leading to discontinuation in the Control arm were acute myocardial infarction and dermatitis (each occurring in 1 patient [3.4%]) and in the BTH1677 arm were atrial flutter, cardiac failure acute, tachyarrhythmia, chills, anaphylactic shock, paresthesia, bronchospasm, hemoptysis, pleural effusion, pulmonary embolism, and pulmonary hemorrhage (each occurring in 1 patient [1.7% each]) and hypersensitivity (occurring in 2 patients [3.4%]).

**Table 3:** Overview of safety outcomes

Adverse Events (AEs), n (%)	BTH1677 (*N* = 59)	Control (*N* = 29)
Any AE	59 (100.0)	29 (100.0)
NCI/CTCAE Grade 3 or 4 AEs	46 (78.0)	25 (86.2)
Serious AEs	37 (62.7)	12 (41.4)
BTH1677-related AEs		
Probably related	26 (44.1)	NA
Possibly related	17 (28.8)	NA
AEs leading to treatment discontinuation	13 (22.0)	2 (6.9)

*AE* adverse events, *NCI/CTCAE* National Cancer Institute Common Terminology Criteria for Adverse Events, *NA* not applicable

All AEs occurring in ≥10% of patients in either the BTH1677 arm or Control arm are presented in Table 4. As expected from the backbone therapy of cetuximab, carboplatin and paclitaxel, skin, hematological, gastrointestinal and neurological AEs occurred frequently in both groups. The difference in specific AE incidence between treatment groups was generally less than 10%, with the exception of neutropenia, anemia, constipation, pyrexia, white blood cell count decreased, paresthesia, peripheral sensory neuropathy, cough, dyspnea, dysphonia, and rash, all of which occurred at a ≥ 10% lower incidence in the BTH1677 arm vs the Control arm. Only 1 AE, skin fissures, occurred at ≥10% higher incidence in the BTH1677 arm vs the Control arm (18.6% vs 6.9%).

**Table 4:** Any grade adverse events occurring in ≥10% of patients and the incidence of these that were grade 3 or 4 within indicated categories (Safety population)

	BTH1677 (*N* = 59)		Control (*N* = 29)	
Adverse Events (AEs), n (%)	All AEs	Grade 3 or Grade 4 AEs	All AEs	Grade 3 or Grade 4 AEs
Number of patients with at least 1 AE	59 (100.0)	46 (78.0)	29 (100.0)	25 (86.2)
Blood and lymphatic system disorders				
Neutropenia	22 (37.3)	19 (32.2)	15 (51.7)	14 (48.3)
Leukopenia	13 (22.0)	9 (15.3)	9 (31.0)	9 (31.0)
Anemia	5 (8.5)	3 (5.1)	8 (27.6)	2 (6.9)
Thrombocytopenia	6 (10.2)	2 (3.4)	5 (17.2)	2 (6.9)
Gastrointestinal disorders				
Nausea	25 (42.4)	0	12 (41.4)	1 (3.4)
Diarrhea	24 (40.7)	0	9 (31.0)	3 (10.3)
Constipation	11 (18.6)	0	10 (34.5)	0
Vomiting	11 (18.6)	0	5 (17.2)	0
Abdominal pain	6 (10.2)	2 (3.4)	4 (13.8)	0
Stomatitis	4 (6.8)	0	4 (13.8)	0
General disorders and administration site conditions				
Fatigue	30 (50.8)	4 (6.8)	17 (58.6)	0
Mucosal inflammation	13 (22.0)	0	6 (20.7)	1 (3.4)
Chest pain	10 (16.9)	0	5 (17.2)	1 (3.4)
Chills	9 (15.3)	2 (3.4)	4 (13.8)	1 (3.4)
Pyrexia	4 (6.8)	0	8 (27.6)	1 (3.4)
Edema peripheral	5 (8.5)	0	5 (17.2)	0
Asthenia	3 (5.1)	0	4 (13.8)	1 (3.4)
Chest discomfort	1 (1.7)	0	3 (10.3)	0
Immune disorders				
Hypersensitivity	4 (6.8)	2 (3.4)	3 (10.3)	0
Infections and infestations				
Nasopharyngitis	6 (10.2)	0	3 (10.3)	0
Paronychia	6 (10.2)	0	2 (6.9)	0
Investigations				
White blood cell count decreased	0	0	3 (10.3)	2 (6.9)
Metabolism and nutritional disorders				
Decreased appetite	13 (22.0)	1 (1.7)	7 (24.1)	1 (3.4)
Hypokalaemia	6 (10.2)	3 (5.1)	3 (10.3)	1 (3.4)
Hypomagnesemia	6 (10.2)	1 (1.7)	2 (6.9)	0
Musculoskeletal and connective tissue disorders				
Myalgia	11 (18.6)	0	6 (20.7)	1 (3.4)
Pain in extremity	10 (16.9)	1 (1.7)	5 (17.2)	0
Bone pain	6 (10.2)	0	5 (17.2)	0
Arthralgia	6 (10.2)	0	4 (13.8)	0
Back pain	5 (8.5)	0	3 (10.3)	1 (3.4)
Neoplasms benign, malignant and unspecified (including cysts and polyps)				
Tumor pain	2 (3.4)	0	3 (10.3)	0
Nervous system disorders				
Polyneuropathy	16 (27.1)	4 (6.8)	9 (31.0)	2 (6.9)
Paresthesia	7 (11.9)	0	9 (31.0)	0
Dizziness	8 (13.6)	0	6 (20.7)	0
Headache	7 (11.9)	0	4 (13.8)	0
Dysgeusia	5 (8.5)	0	5 (17.2)	0
Peripheral sensory neuropathy	0	0	3 (10.3)	0
Psychiatric disorders				
Insomnia	7 (11.9)	0	5 (17.2)	0
Respiratory, thoracic and mediastinal disorders				
Cough	12 (20.3)	0	11 (37.9)	0
Dyspnea	12 (20.3)	1 (1.7)	10 (34.5)	1 (3.4)
Dysphonia	6 (10.2)	1 (1.7)	6 (20.7)	0
Epistaxis	7 (11.9)	0	5 (17.2)	0
Pleural effusion	7 (11.9)	3 (5.1)	3 (10.3)	0
Oropharyngeal pain	3 (5.1)	0	3 (10.3)	0
Skin and subcutaneous tissue disorders				
Rash	28 (47.5)	2 (3.4)	19 (65.5)	3 (10.3)
Alopecia	22 (37.3)	0	13 (44.8)	0
Dermatitis	12 (20.3)	0	5 (17.2)	0
Pruritus	9 (15.3)	1 (1.7)	4 (13.8)	1 (3.4)
Skin fissures	11 (18.6)	0	2 (6.9)	0
Dry skin	6 (10.2)	0	3 (10.3)	0

For the AEs occurring in ≥10% of patients, the incidence occurring at Grade 3 or Grade 4 is also shown in Table 4. The most common Grade 3 or Grade 4 AEs were hematological, of which a lower incidence was again seen in the BTH1677 arm vs the Control arm. Grade 3 or Grade 4 AEs seen exclusively in the BTH1677 arm were abdominal pain (3.4%), fatigue (6.8%), hypersensitivity (3.4%), decreased hemoglobin (6.8%), hypomagnesemia (1.7%), pain in extremity (1.7%), dysphonia (1.7%), and pleural effusion (5.1%). Grade 3 or Grade 4 AEs seen exclusively in the Control arm were nausea (3.4%), diarrhea (10.3%), mucosal inflammation (3.4%), chest pain (3.4%), pyrexia (3.4%), asthenia (3.4%), white blood cell count decreased (6.9%), myalgia (3.4%), and back pain (3.4%). Instances of these events were low, generally occurring in only 1–2 patients. Evaluation of all AEs and Grade 3 or Grade 4 AEs by ABA biomarker status did not demonstrate differences between groups (data not shown).

Seven deaths (6 in the BTH1677 arm; 1 in the Control arm) were reported in the treatment phase or within 30 days of the last dose of study medication. Four of the six deaths in the BTH1677 arm were due to disease progression; the other two were due to neutropenia/sepsis/acute renal failure (possibly related to carboplatin and paclitaxel and unlikely related to BTH1677 and cetuximab) and pleural effusion caused by previously broken ribs (unlikely related to any treatment). The one death that occurred in the Control arm was due to disease progression.

### BTH1677 pharmacokinetics

Overall, serum BTH1677 concentration-time profiles were well characterized and consistent in Cycle 1 and Cycle 3. All serum concentrations of BTH1677 were above the limit of quantitation (4.7 ng/mL) in both cycles and mean concentrations declined in a multi-exponential manner over time (data not shown). Table 5 summarizes the BTH1677 PK parameters from Cycle 1/Day 1 (*N* = 52) and Cycle 3/Day 1 (*N* = 36). The difference between the *N* for the two cycles largely reflected patients discontinuing the study between Cycle 1 and Cycle 3. Geometric mean AUC_0–24_ values of BTH1677 were similar in Cycle 1 (362 μg•hr./mL) and Cycle 3 (396 μg•hr./mL). Geometric mean C_max_ values (44.3 μg/mL and 47.8 μg/mL) and median t_max_ values (2.25 h and 2.40 h) were also similar in both Cycle 1 and Cycle 3, respectively. Cross-cycle comparison of all other PK parameters (AUC_0-last_, AUC_0-∞_, CL, and t_1/2_) should be interpreted with caution since there was a difference in the blood sampling interval between cycles (Cycle 1: 168 h and Cycle 3: 24 h). The apparent shorter elimination half-life of BTH1677 in Cycle 3 relative to Cycle 1 (8.46 h and 19.1 h, respectively) is likely to be representative of a distribution phase rather than an elimination phase. Overall, the data suggest that exposure to BTH1677 was consistent in both cycles. BTH1677 levels assessed weekly before dosing through Day 1 Cycle 3, revealed no meaningful accumulation of BTH1677 with weekly dosing. Mean clearance of BTH1677 on Day 1 of Cycles 1 and 3 (0.477 L/h and 0.696 L/h, respectively) were consistent with that reported in healthy subjects (0.441 L/h – 0.619 L/h) [34].

**Table 5:** Summary of BTH1677 pharmacokinetics parameters

Parameters	Geometric Mean (CV%)	
	BTH1677	
	Cycle 1/Day 1	Cycle 3/Day 1
N	52	36
AUC_0–last_ (μg•hr./mL)	605 (55.3)	396 (38.1)
AUC_0–24_ (μg•hr./mL)	362 (35.2)	396 (34.8)
AUC_0–∞_ (μg•hr./mL)	621 (53.1)	416 (30.4)
C_max_ (μg/mL)	44.3 (34.9)	47.8 (37.3)
CL (L/h)	0.477 (46.4)	0.696 (32.1)
t_1/2_ (hr)	19.1 (42.8)	8.46 (23.2)
t_max_ (hr)*	2.25 (1.97, 4.33)	2.40 (1.93, 4.17)
V_ss_ (L)	6.60 (35.5)	6.48 (39.9)

*N* number of patients, *CV* coefficient of variation, *AUC*_*(0-last)*_ area under the plasma concentration-time curve from time 0 to the time of the last measurable concentration, *AUC*_*0–24*_ area under the plasma concentration-time curve from time 0 to 24 h, *AUC*_*0–∞*_ area under the plasma concentration-time curve from time 0 to infinity, *C*_*max*_ maximum plasma concentration, *CL* systemic clearance; t_1/2_, elimination half-life, *t*_*max*_ time of maximum concentration, *V*_*ss*_ volume of distribution at steady-state

*Median (range)

Exploratory analyses of PK parameters by biomarker status revealed no difference between biomarker-positive vs biomarker-negative patients (data not shown).

## Discussion

BTH1677 is a novel PAMP being developed for the treatment of cancer in combination with tumor-targeted antibodies, as well as anti-angiogenic and checkpoint inhibitor antibodies. A previous phase Ib/II study in second- to third-line mCRC patients evaluated BTH1677 combined with cetuximab and showed good tolerability with promising efficacy [34–37]. This randomized, open-label, phase II study demonstrated that BTH1677 in combination with cetuximab, carboplatin, and paclitaxel numerically improved the ORR in patients with previously untreated, advanced NSCLC by 13% (*p* = 0.2895; central review) to 24% (*p* = 0.0468; investigator review) beyond the ORR observed in Control patients. No meaningful changes were observed in other tumor-associated assessments or survival. Subject numbers were small for time-to-event assessments and not all randomized subjects were included in the primary efficacy populations. Furthermore, large differences in the percentage of patients censored for some of these assessments in the investigator (~20%) vs central (~50%) reviews no doubt further clouded an ability to see consistent meaningful changes.

The population enrolled in this study was similar to the large BMS099 study (*N* = 676) evaluating cetuximab and carboplatin/taxane vs carboplatin/taxane alone as first-line treatment in advanced NSCLC patients that were not selected for EGFR expression or *KRAS* gene status [13]. Our Control ORRs of 23.1% (both investigator and central reviews) compare similarly to the ORRs of 27.5% (investigator review) and 25.7% (central review) in the BMS099 cetuximab and carboplatin/taxane arm. In light of our Control ORR responding as expected, the improved 47.8% (investigator review) and 36.6% (central review) ORRs observed with the addition of BTH1677 to the backbone therapy does suggest further benefit from the addition of BTH1677. However, whether improved ORR will translate into benefit in more clinically relevant endpoints such as PFS or OS would require larger studies powered for such endpoints. Even in the large BMS099 study, improved ORR did not translate into improved PFS or OS. In the larger FLEX study (*N* = 1125), evaluating cetuximab and cisplatin/vinorelbine vs cisplatin/vinorelbine alone as first-line treatment in selected EGFR-expressing advanced NSCLC patients [14], the slight improvement in ORR (36% vs 29%) was associated with a statistically significant improvement in OS, but the effect was very modest (11.3 vs 10.1 months; HR 0.871; *p* = 0.044).

Interestingly, our findings indicated that ABA level may be useful as a predictive biomarker for response to BTH1677. BTH1677 biomarker-positive patients exhibited better OS than biomarker-negative patients. However, no serum was available from Control patients for ABA analysis and, therefore, key comparisons of BTH1677 biomarker-positive and biomarker-negative responses to Control biomarker-positive and biomarker-negative responses could not be performed. Hence, the possibility that ABA level may be prognostic for better responders vs predictive of a BTH1677-specific effect cannot be ruled out. Given the critical role of ABA in facilitating the binding of BTH1677 to immune cells [18–22, 39–42] the former seems unlikely; however, controlled studies are needed to further assess the true contribution of ABA in response to BTH1677, as well as to refine the most appropriate ABA thresholds for defining biomarker-positive and biomarker-negative patients in the cancer population.

In terms of safety, BTH1677 combination therapy was well tolerated among patients, which was consistent with previous experience using BTH1677 in combination with cetuximab in mCRC patients [34–37]. Overall, most AEs were mild or moderate in severity. Only skin fissures occurred at a 10% greater incidence in the BTH1677 arm than in the Control arm (18.6% vs 6.9%).

PK parameters were consistent with those previously observed in healthy volunteers [34], suggesting that neither cetuximab nor the carboplatin and paclitaxel therapy affected the PK of BTH1677.

In conclusion, BTH1677 in combination with cetuximab and concomitant carboplatin and paclitaxel improved ORR and was well tolerated in patients with previously untreated, advanced NSCLC. At the time this study was initiated, cetuximab was undergoing regulatory review for first-line use in late-stage NSCLC patients, but ultimately never received approval. Hence, although the treatment regimen used here will not proceed to further evaluation, the results of this study support the concept of improved efficacy with the addition of BTH1677 to antibody therapy. The NSCLC study run in parallel with this study, that evaluated BTH1677 combined with bevacizumab/carboplatin/paclitaxel in non-squamous late-stage NSCLC patients, also demonstrated promising results [43], and a NSCLC study of BTH1677 in combination with pembrolizumab is ongoing (ClinicalTrials.gov NCT03003468). This novel therapeutic also continues to be investigated in combination with antibody therapies in additional oncology clinical trials that also include further investigation into the possible role of ABA as a predictive biomarker for response to BTH1677.
